# Loop-mediated isothermal amplification applied to filarial parasites detection in the mosquito vectors: *Dirofilaria immitis *as a study model

**DOI:** 10.1186/1756-3305-2-15

**Published:** 2009-03-15

**Authors:** Hiroka Aonuma, Aya Yoshimura, Namal Perera, Naoaki Shinzawa, Hironori Bando, Sugao Oshiro, Bryce Nelson, Shinya Fukumoto, Hirotaka Kanuka

**Affiliations:** 1 National Research Center for Protozoan Diseases, Obihiro University of Agriculture and Veterinary Medicine, Inada-cho, Obihiro, Hokkaido 080-8555, Japan; 2 Medical Research Institute, Colombo 08, Sri Lanka; 3 Department of Genetics, Graduate School of Pharmaceutical Science, University of Tokyo, Hongo, Bunkyo-ku, Tokyo 113-0033, Japan; 4 Yanbaru Animal Clinic, Nago, Okinawa 905-0019, Japan

## Abstract

**Background:**

Despite recent advances in our understanding of the basic biology behind transmission of zoonotic infectious diseases harbored by arthropod vectors these diseases remain threatening public health concerns. For effective control of vector and treatment, precise sampling indicating the prevalence of such diseases is essential. With an aim to develop a quick and simple method to survey zoonotic pathogen-transmitting vectors, LAMP (loop-mediated isothermal amplification) was applied to the detection of filarial parasites using a filarial parasite-transmitting experimental model that included one of the mosquito vectors, *Aedes aegypti*, and the canine heartworm, *Dirofilaria immitis*.

**Results:**

LAMP reactions amplifying the cytochrome oxidase subunit I gene demonstrated high sensitivity when a single purified *D. immitis *microfilaria was detected. Importantly, the robustness of the LAMP reaction was revealed upon identification of an infected mosquito carrying just a single parasite, a level easily overlooked using conventional microscopic analysis. Furthermore, successful detection of *D. immitis *in wild-caught mosquitoes demonstrated its applicability to field surveys.

**Conclusion:**

Due to its simplicity, sensitivity, and reliability, LAMP is suggested as an appropriate diagnostic method for routine diagnosis of mosquito vectors carrying filarial parasites. This method can be applied to the survey of not only canine filariasis but also lymphatic filariasis, another major public health problem. Therefore, this method offers great promise as a useful diagnostic method for filarial parasite detection in endemic filariasis regions.

## Background

For years, vector-borne zoonotic infectious diseases have had profound debilitating effects on humans. It has recently been revealed that several arthropod vectors including mosquitoes act as "bridge" vectors by transmitting pathogens from animals to humans and vice versa [1]. Human clinical cases of diseases, such as dirofilariasis, babesiosis, and leishmaniases, all caused by parasites transmitted by arthropod vectors from animals, have been reported to be on the rise [2-6]. Infection with these diseases have affects not only on humans but also on domestic and wild animals that can serve as potential reservoirs by hosting pathogens long-term despite being asymptomatic. Because eradication of animal reservoirs has been ethically rejected, surveillance and control of arthropod vectors must be central to programs aimed at elimination of vector-borne zoonotic diseases [5].

*Dirofilaria *species, including *D. immitis *and *D. repens*, are particularly important pathogens due to the fact that they induce serious symptoms in domestic animals, especially dogs. Adult worms in infected canines release microfilariae in their blood stream that are then ingested by mosquitoes during blood feeding. Mosquitoes then harbor these parasites until they reach the third larval stage (L3) at which point transmission back to canines is possible. Several common mosquito species are capable of transmission including, occasionally, to humans where, despite acting as a "dead end" host for the *Dirofilaria *parasites, symptoms such as coughing and chest pain are induced [2]. Human clinical cases of zoonotic infection of *Dirofilaria *are increasing in various countries [2-4] leading to frequent misdiagnosis as lung tumors or tuberculosis rather than pulmonary dirofilariasis [3,4]. Further complicating the situation is that analysis of many clinical cases involving infected humans revealed a lack of any exposure to infected domestic dogs suggesting transmission occurred through wild animals [2]. Indeed, it has been reported that wild animals including raccoons, dogs, wolves, dingoes, coyotes, and foxes are capable of infection with *Dirofilaria *species thereby serving as reservoirs [7-12]. Therefore, control of mosquitoes based on precise surveillance of *Dirofilaria *species is essential for preventing infection of both domestic animals and humans with these parasites.

Surveying of filarial parasites has long depended on microscopic examination of dissected mosquitoes or blood films. Unfortunately, this method requires a practiced-eye and oversight often occurs in specific stages where the parasite is difficult to recognize. Additionally, microscopy is laborious and time-consuming thereby impeding routine monitoring of large-scale control programs. Application of PCR (polymerase chain reaction) has been considered as a more accurate and practical diagnostic alternative leading recently to the development of several methods [13-17]. Despite its outstanding sensitivity and specificity, drawbacks remain due to the need for expensive equipment and trained technicians. Hence to reveal precisely the distribution of risk to filarial diseases novel diagnostic methods that are simple, rapid, sensitive, and reliable are required. In consideration of these points we propose a novel surveillance strategy.

Loop-mediated isothermal amplification (LAMP) is a novel DNA amplification method that allows reactions to occur under isothermal conditions [18]; the *Bst *DNA polymerase can synthesize a new strand of DNA while simultaneously displacing the complementary strand thereby enabling DNA amplification at a single temperature with a single enzyme. Four primers are required for the LAMP reaction: FIP, BIP, F3, and B3. F3 and B3 contribute to the formation of a stem-loop structure while the other two primers, FIP and BIP, designed complementary to the inner sequence of the stem-loop structure, are used to amplify the target sequence, thus providing a higher specificity to the reaction than conventional PCR methods. Another advantage using LAMP is based on the fact that the amplification from stem-loop structures leads to accumulation of large amounts of products of varying lengths ultimately making detection of amplified DNA much easier. Furthermore, the by-product of the reaction, magnesium pyrophosphate, is a white-colored precipitate easily seen by the naked eye [19].

Recently, wide applicability of LAMP in the detection of parasites such as *Trypanosoma*, *Babesia*, and *Plasmodium *has been demonstrated [20-23]. Additionally, the usefulness of LAMP has been applied to the identification of genus- and species-specific parasites [24,25]. However, application of LAMP to survey vectors has been largely neglected despite its promise. Due to the fact that a novel applicable method to survey filarial-carrying mosquitoes is still demanded, we propose the application of LAMP for detection of filarial parasites within vectors. Mosquitoes carrying one or more parasites have the ability to infect vertebrate hosts during blood feeding. This implies that all mosquitoes containing just a single parasite need to be identified unfailingly by LAMP in order to ensure a precise and practical survey. In this study, we applied LAMP in the detection of *D. immitis*, both within mosquito vectors using *Ae. aegypti *as laboratory model [26] in addition to field samples in order to demonstrate its potential as an important diagnostic tool to be coupled with ongoing vector control measures.

## Method

### Preparation of parasite and infected mosquitoes

Adult *Dirofilaria immitis *were isolated from an infected dog and cultured in RPMI 1640 media. Microfilariae were paralyzed by cooling to 4°C and collected by centrifugation at 90 to 130 × g for 5 to 10 minutes. Isolated microfilariae were counted on a cytometer and stored at -20°C until DNA extraction. To evaluate usability of the LAMP method as a practical vector-diagnostic method, LAMP-based detection of *D. immitis *within mosquitoes was carried out. *Aedes aegypti *was fed with *Dirofilaria*-infected canine blood by membrane feeding. Briefly, Parafilm^® ^(Pechiney Plastic Packaging, Inc., Illinois, USA) was fitted under the culture flask containing warm water and filled with infected canine blood. The membrane feeder was set over the netted mosquito cup and mosquitoes were allowed to feed on infected blood for 1 hour. Infected mosquitoes were kept at 27°C until 8 days post infection when *Dirofilaria *in mosquitoes reached the L2 or L3 stage. Each Malpighian tubules together with the carcass of the infected mosquito was dissected in ice-cold PBS at day 8, microscopically analyzed at 50-fold magnification to count the number of parasites, and stored at -20°C until DNA extraction. *Dirofilaria *L2 or L3 larvae removed from Malpighian tubules were also stored for use as positive control of LAMP.

### Mosquito collection

Wild mosquitoes were collected in Nago, Okinawa, Japan in August 2008. Mosquitoes were caught either with CDC traps (John W. Hock Co., Florida, USA) using CO_2 _or by sweeping nets at locations near domestic dogs. Collected mosquito species were identified based on keys according to description and illustrations [27].

### DNA extraction

Genomic DNA of *D. immitis *larvae and infected mosquitoes was extracted as follows: *D. immitis *larvae and infected mosquitoes were collected, homogenized with a plastic homogenizer in 100 μl of Buffer A (0.1 M Tris (pH 9.0), 0.1 M EDTA, 1% SDS, and 0.5% DEPC), and incubated for 30 minutes at 70°C. 22.4 μl of 5 M KoAc was added to the mixture and incubated for 30 minutes on ice. Supernatant was collected by centrifugation at 20400 × g for 15 min at 4°C and mixed with 45 μl of isopropanol. Precipitated DNA was collected after centrifugation at 20400 × g for 20 min at 4°C, rinsed with 70% ethanol, and dried. Each DNA pellet was diluted in TE to achieve a concentration such that 1 μl of solution would contain DNA from 1 × 10^4 ^microfilariae (152 ng) or one-fifth of a mosquito. 1 μl of each DNA solution was then used as a template for LAMP reaction.

### LAMP reactions

**Figure 1:**
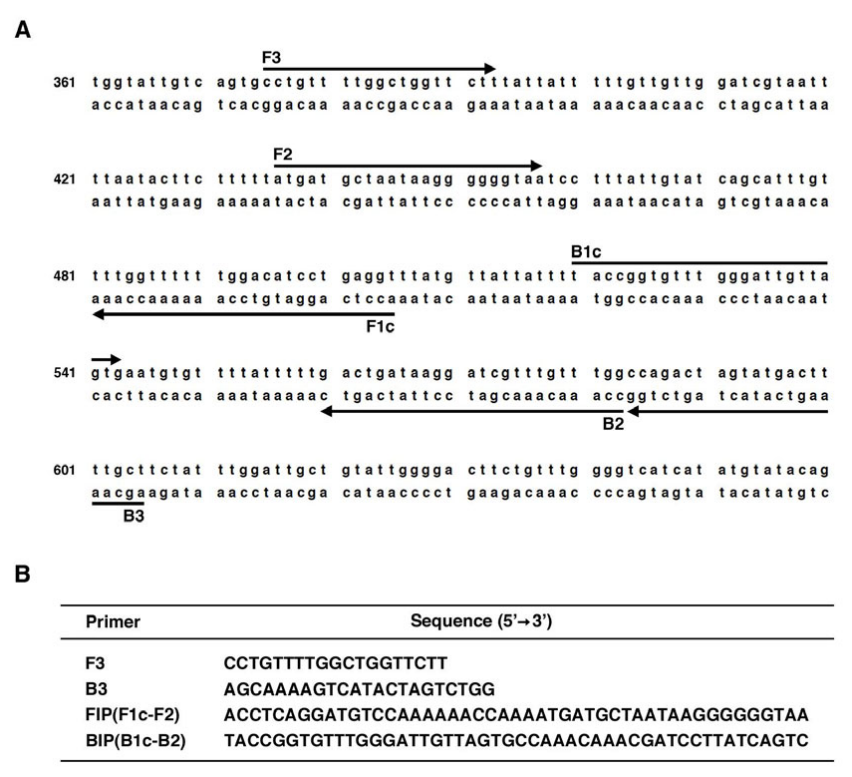
**LAMP primer set targeting *D. immitis *cytochrome oxidase subunit I gene**. (A) Partial sequence of *D. immitis *cytochrome oxidase subunit I gene and location of four primers, FIP (F1c-F2), BIP (B1c-B2), F3, and B3. Arrow indicates the direction of extension. Numbers on the left indicate the nucleotide position. (B) Sequence of primers for LAMP reaction.

Specific primers for LAMP reactions were designed against the *D. immitis *cytochrome oxidase subunit I gene (GenBank: EU169124). The locus and sequence of each primer (F3, B3, FIP, BIP) in this gene are shown in Fig. 1. The LAMP reaction was performed as per manufacture's instructions (Eiken Chemical Co., Ltd., Tokyo, Japan). Briefly, the reaction was performed in 12.5 μl of reaction mixture containing 1 μl of extracted DNA solution, 2.5 pmol of each F3 and B3 primers, 20 pmol of each FIP and BIP primers, 6.25 μl of 2× Reaction Mix., and 0.5 μl of *Bst *DNA polymerase. The reaction mixture was incubated at 63°C for 70 min using a Loopamp Realtime Turbidimeter (LA-200; Eiken Chemical Co., Ltd., Tokyo, Japan) and terminated by incubation at 95°C for 2 minutes.

### Analysis of LAMP products

Amplified DNA in the LAMP reaction causes turbidity due to the accumulation of magnesium pyrophosphate, a by-product of the reaction. Turbidity was monitored using Loopamp Realtime Turbidimeter in addition to the naked eye. All LAMP products were electrophoresed on 2% agarose gels, stained with ethidium bromide and visualized under UV light.

### DNA sequencing of LAMP products

LAMP reaction products forming structures with stem-loops at each end were collected from agarose gels after electrophoresis and purified by QIAEX II Gel extraction Kit (QIAGEN, Dusseldorf, Germany) following manufacture's instructions. Sequences were directly determined with FIP and BIP primers using ABI PRISM 3100 Genetic Analyzer (Applied Biosystems, CA, USA) and analyzed using BLAST.

## Results

### Sensitivity of LAMP in the detection of *Dirofilariaimmitis*.

Primers for *D. immitis *detection were designed to target the *D. immitis *cytochrome oxidase subunit I gene due to availability of sequence data in GenBank (EU169124) (Fig. 1A and 1B). Initial tests examining specificity and sensitivity of this primer set were performed using serial dilutions of *D. immitis *DNA prepared from cultured microfilariae. Optimization of LAMP reaction conditions (temperature and time) using the described primer set revealed ideal settings to be 63°C and 70 minutes (data not shown). Sequencing of these LAMP products showed that amplified DNA were of *D. immitis *cytochrome oxidase subunit I gene (data not shown).

**Figure 2:**
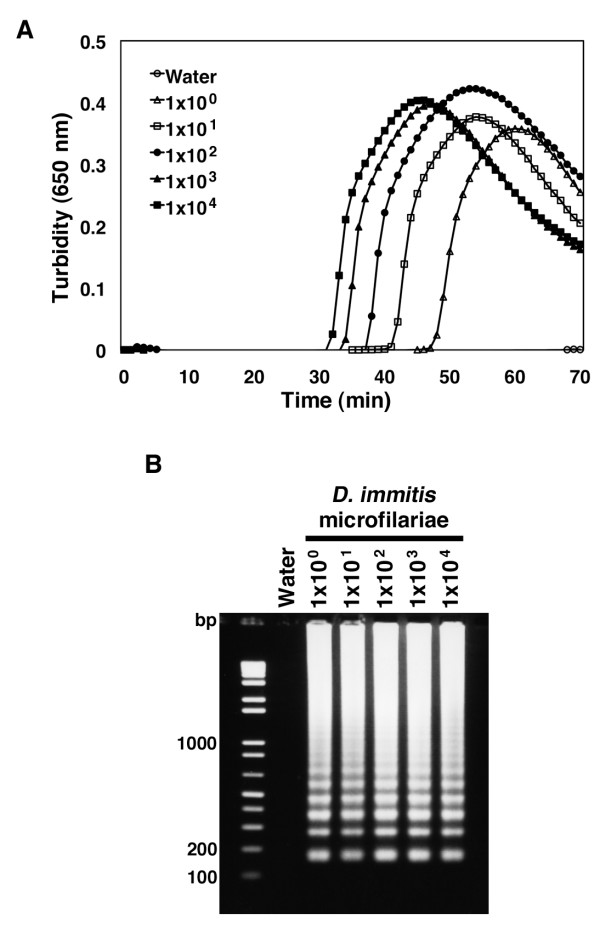
**Kinetics of LAMP for *D. immitis *microfilariae**. (A) Amplification of target sequence with primer set monitored by real-time turbidimeter (turbidity at 650 nm). (B) Detection of reaction products by LAMP with genomic DNA from *D. immitis *microfilariae. 1 μl of reaction mixture was run on a 2% agarose gel. Amplified products were detectable with any samples containing genomic DNA corresponding to the amount of one or more microfilariae. Numbers on the left indicate migration of molecular weight marker (bp).

Having determined the experimental conditions, the sensitivity of LAMP reactions in the detection of the *D. immitis *microfilariae was tested. *D. immitis *microfilariae DNA (equivalent to 1 × 10^0^, 1 × 10^1^, 1 × 10^2^, 1 × 10^3^, and 1 × 10^4 ^parasites) obtained from cultured adult worms was used as a template for LAMP reactions and yielded an amplified product approximately 30 minutes after incubation using DNA from 1 × 10^4 ^parasites (152 ng) (Fig. 2A). Importantly, a product could be detected from samples containing just 1 × 10^0 ^microfilaria while control samples remained negative (Fig. 2A). All samples containing DNA of microfilariae showed white precipitates detected by naked eye (data not shown). In agreement with turbidity analysis and eye-observation, gel electrophoresis also showed that one microfilaria is sufficient for LAMP detection (Fig. 2B). These data indicate that LAMP reactions using the primer set designed against the *D. immitis *cytochrome oxidase subunit I gene is able to identify purified parasites collected from both the blood and vector stages down to levels of a single microfilaria parasite.

### Evaluation of LAMP for diagnosis of *D. immitis*-carrying mosquitoes.

**Figure 3:**
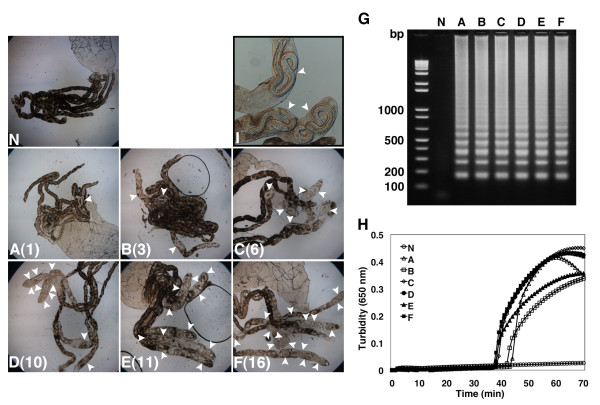
**Evaluation of LAMP by detecting L2 stage *D. immitis *in mosquitoes**. *D. immitis *in L2 stage were counted under microscopy prior to DNA extraction. (A-F) Mosquito Malpighian tubules infected with *D. immitis *in L2 stage. Figure in parentheses indicates the number of *D. immitis*. (N) Malpighian tubules from intact mosquito. White arrowheads indicate *D. immitis *in mosquito Malpighian tubules. (G and H) LAMP detection of *D. immitis *by electrophoresis (G) and real-time turbidimeter (H) with genomic DNA from mosquitoes corresponding to (N)-(F) shown left. Numbers on the left indicate migration of molecular weight marker (bp). (I) An example of mosquito Malpighian tubules infected with *D. immitis *L2 larvae at 200-fold magnification.

One of the field mosquito vectors of *D. immitis*, *Ae. aegypti *[28], was employed as laboratory model [26]. In an experiment using 6 independent mosquito samples, the number of L2 parasites ranged from 1 to 16 (Fig. 3A–3F). After microscopic observation, each Malpighian tubules was collected together with its carcass before being subjected to DNA extraction to be used as a template for LAMP reactions. The amplified product of each reaction mixture was determined by a combination of electrophoresis and a real-time turbidimeter revealing a detection profile correlated with L2 parasite numbers (Fig. 3G and 3H). Consistent with these results, white precipitates, which indicates LAMP-dependent DNA amplification, were produced in all reaction mixtures of infected samples (data not shown). It is particularly worth noting that even a single L2 parasite within a mosquito was successfully detected by the LAMP reaction (Fig. 3G and 3H).

### LAMP-based identification of wild-caught *D. immitis*-carrying mosquitoes.

**Figure 4:**
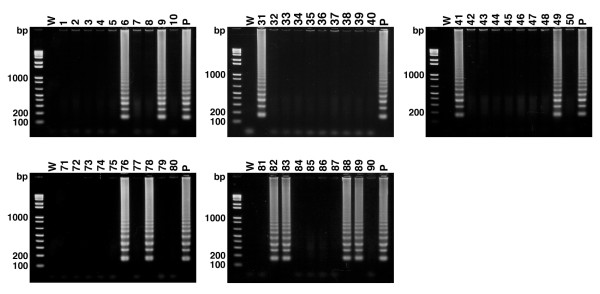
**LAMP-based identification of wild-caught *D. immitis*-carrying mosquitoes**. Detection of *D. immitis *by LAMP with genomic DNA from individually wild-caught mosquitoes within 5 positive groups screened initially. 11 among 120 mosquitoes showed positive signal indicating carrying *D. immitis*. Water served as a negative control (W) and pure DNA from *D. immitis*L3 served as a positive control (P).

To evaluate whether the LAMP method is appropriate for diagnosis of *D. immitis*-carrying mosquitoes, we applied this technique to intact wild mosquitoes caught in the field. Mosquitoes were collected at several locations in Okinawa, Japan, species were determined (72 *Aedes albopictus*, 43 *Armigeres subalbatus*, 2 *Culex pipiens*, 2 *Culex vishunui comp.*, and 1 *Aedes aureostriatus*), and DNA was extracted. For initial screening, DNA from 10 mosquitoes were pooled and used for each LAMP reaction. LAMP analysis revealed that 5 out of 12 groups reacted positively indicating one or more infected mosquitoes to be contained within those groups (data not shown). Individual mosquitoes from positive groups were then tested for the presence of *Dirofilaria *parasites. Use of LAMP revealed each positive group of 10 mosquitoes to contain 1 to 4 infected mosquitoes (totally 10 *Ae. albopictus *and 1 *Ar. subalbatus*) (Fig. 4). The amplified products of LAMP reactions were sequenced and determined to be of *D. immitis *(data not shown).

## Discussion

In this study, we demonstrated the detection of filarial parasites within vector mosquitoes using the LAMP method. Elimination of zoonotic infectious diseases has been identified as crucial to public health particularly in light of the recent emergence and re-emergence of diseases such as West Nile virus, malaria, dengue fever, Japanese encephalitis and novel clinical cases of infection of humans with diseases previously unknown to infect humans. Many of these diseases require arthropod vectors, therefore, surveillance-based control of arthropods is critical in the management of such vector-borne diseases.

The ability of LAMP to detect just a single microfilaria suggests the applicability of this method for clinical cases with extremely low microfilaremia. Complicating their detection at the vector stage is potential contamination with mosquito debris, an issue of particular concern for PCR based detection methods. However, this appears to be overcome by LAMP as shown by sensitivity of detection down to levels of a single parasite within a whole mosquito. Furthermore, sequencing of LAMP products showed specific amplification of *D. immitis *DNA, indicating the high specificity of this method. Considering infected mosquitoes typically carry anywhere from 1 to 40 parasites it appears this method is sufficient to address the need for a reliable and sensitive diagnostic method that is completed within 60 minutes.

Human lymphatic filariasis remains a major public health problem reported to put more than a billion people in 83 endemic countries at risk of infection [29] with *Wuchereria bancrofti *and *Brugia malayi *or *B. timori *being the most common parasites [30]. In 1997, WHO called for the elimination of lymphatic filariasis [31] and The Global Programme to Eliminate Lymphatic Filariasis (GPELF) has been given the task of eliminating filariasis by 2020 [32]. To achieve this elimination-goal, emphasis was placed on the use of the newest, most cost-effective diagnostic and mapping tools available [30]. These tools should also have the added characteristics of being sensitive and specific. The cost of LAMP reaction is comparative to PCR reaction, and it can be carried out with lower cost due to no requirement for a thermal cycler but only warm water. Additionally, we have already confirmed that LAMP can be well performed using reduced reaction mixture to half volume with detection by naked eye and electrophoresis (data not shown), suggesting the possibility of further reduction of cost. The applicability of LAMP to field-surveys, and hence as a diagnostic and mapping tool, was demonstrated by performing diagnosis of wild-caught mosquitoes. Specifically, *Dirofilaria*-carrying mosquitoes collected from an endemic area were identified from a pool of infected and non-infected mosquitoes via LAMP reactions. Taken together, the LAMP method appears ideal to fit the criteria laid out for the eradication of filariasis through careful and reliable monitoring of parasites, yet further studies to test this method for the detection of the parasites species responsible of lymphatic filariasis are needed.

## Conclusion

Our successful detection of parasites demonstrated the applicability of LAMP in the diagnosis of filarial parasites-carrying mosquitoes using not only a laboratory model but also field samples. Due to its isothermal reaction conditions and simple diagnostic output, LAMP can be easily combined with typical field collections of vectors to survey pathogens *in situ*; indeed, only warm water is required to perform this assay. Though we used *D. immitis *as a model, our method is also applicable to diagnosis of other vector-borne filarial diseases such as *W. bancrofti*, *B. malayi*, and *D. repens*. With these features this method offers great promise to achieve a useful method for surveying filarial parasites in regions where filariasis is endemic.

## Competing interests

The authors declare that they have no competing interests.

## Authors' contributions

HA conceived the study, performed the experiments, and wrote the manuscript. AY prepared filarial parasites and infected mosquitoes. NP assisted with determination of reaction conditions. NS and HB helped collection of wild mosquitoes. SO arranged collection of mosquito. BN clarified the manuscript. SF conceived and supervised the study. HK supervised the study. All authors read and approved the final manuscript.
